# Mapping the Greek Wine Supply Chain: A Proposed Research Framework

**DOI:** 10.3390/foods10112859

**Published:** 2021-11-18

**Authors:** Foivos Anastasiadis, Maria Alebaki

**Affiliations:** 1 Department of Agricultural Economics, School of Agriculture, Aristotle University of Thessaloniki, 54124 Thessaloniki, Greece; 2 Agricultural Economics Research Institute (AGRERI), Hellenic Agricultural Organization DIMITRA, Terma Alkmanos St., 11528 Athens, Greece; mariale@agreri.gr

**Keywords:** food supply chain, agri-food sector, sustainability, value chain

## Abstract

The wine industry involves both the production (i.e., from vine to wine) and consumption of wine (i.e., dining and tourism experiences). This results in a complicated system of intertwined product and services supply chains. Recent studies in the field suggest several key perspectives for future research, such as sustainability, resilience and circular economy. However, the complex nature of the sector which comprises numerous stakeholders and flows (i.e., products—services—information), entailing knotty interactions and interdependencies, makes any research direction questionable in terms of its effectiveness. Therefore, the objective of this study is to provide a tool for designing targeted future research in such multiple environments. We employed an end-to-end mapping approach using data from the Greek wine supply chain, identifying essential insights for a compelling research agenda. The key output is a set of three supply chain maps revealing the structure, processes and actors from all the main angles: basic production, value chain and stakeholders. A synthesis of these maps supports an overall understanding of the sector, unmasking any interaction and hidden information holdups. The study thus aims to offer an integrated research framework that highlights the leading priorities of the Greek wine industry.

## 1. Introduction

Wine remains among the oldest [1] and most popular alcoholic beverages globally [2], representing a dynamic market [3,4] in the economy of both Old- and New- World countries. In 2020, the total surface area planted with vineyards for all purposes (wine and juices, table grapes and raisins) was estimated at 7.3 mha [5,6,7]. In 2020, world wine production—excluding juices and musts—was assessed at 260.0 mhl, slightly increased (+1.0%) compared to the previous year [5,6,7]. Italy, France and Spain account for 53.0% of the global production (49.1, 46.6 and 40.7 mhl, respectively) [3]. According to the latest report [8], EU27 wine production in 2021 is expected to reach 147 mhl, registering a decrease compared to 2020 (165 mhl) [3]. Although the EU27 accounts for 48.0% of the global wine consumption [3], the USA remains the largest wine consuming country in the world [3]. In terms of international trade, the “State of the world vitivinicultural sector in 2020” report [3] renders Italy as the largest exporter in 2020 (20.8 mhl, 20.0% of the global market).

As far as Greece is concerned, wine constitutes a strategic agri-food product for the country’s economy. The long national tradition in viticulture renders wine an indispensable part of the Greek culture for over 4000 years [9]. Currently, there are approximately 1350 wineries (an increase of more than 100.0% over the last decade), 692 of which have the license to produce PDO (Protected Designation of Origin) and PGI (Protected Designation of Indication) wines [6,7]. As Alebaki and Iakovidou [10] underline, the large differentiation of the wines produced in the country (7500 labels) can be attributed to the variety of vineyards (there are 280 indigenous varieties) [6,11]; quality origins; and production methods.

In 2019, the total area under vineyards in Greece reached 63,200 ha (3.05% of the total agricultural area in Greece, while wine grapes account for 1.71% and table grapes for the remaining 1.34%), a decrease since 2009 (70,000 ha). In 2020, Greek wineries produced 2.4 million hundred litres, representing 1.0% of the world wine production. In particular, Greek PDO (Protected Designation of Origin) and PGI (Protected Designation of Indication) wines account for 22.7% of the country’s total wine production, while table wines comprise 67.8%. Domestic consumption reached 1.6 million hl (42.8% decreased compared to 2008/9) [5,6,7]. Moreover, Greece ranked 26th in exports globally in 2019 (87 million US$) [5,6,7,11]. The same year, wine exports and imports reached 79.5 (+34.5% since 2009) and 48.4 (+61.33% since 2009) million Euros respectively [5,6,7].

It is important to be stressed that the relative boom of the wine industry in Greece is partly due to the following reasons: Firstly, in 1969, to fulfil the preconditions to join the European Union, Greece revised its legislative framework for wines. Secondly, in 1988, the use of the term “regional wine” has been approved by the national regulations [10]. These developments have led to a quality improvement of the wines produced and a revival of the country’s wine sector, which—since the early 1990s- has recorded remarkable progress [12,13]. The aforementioned advancements have been further reinforced by the joint actions of wine producers in several wine regions, who have gradually created non-profit associations; Thus, since the pioneer example of Northern Greece in 1993, Peloponnese; Attica; Crete; Central Greece; and the Aegean Islands followed suit [10].

The main objectives of winemakers’ networks included, inter alia, the development of wine tourism [14]. The latter represents an emerging form of tourism that incorporates a wide set of activities and infrastructure. Specifically, wine tourism is -simultaneously- a form of consumer behaviour; a promotion/sales channel for wineries and a wine destination strategy [15]. In a more holistic approach, wine tourism can be viewed as a sub-system of the tourism sector that comprises both tangible and intangible components, as well as multiple resources (human; industrial; environmental; institutional) of the supply and demand [12]. As Alebaki & Ioannides [16] argue: “*Having emerged from the existing symbiotic relationship between agriculture, manufacturing, and the tertiary sector, wine tourism…is particularly exposed to multiple stressors*”.

From a supply chain perspective, this raises the complexity of the wine sector in general, as the variety of products and services (viticulture, winemaking and wine tourism) involve diverse value chains and numerous stakeholders through the entire supply chain. Thus, the environmental, social and economic sustainability of the sector, particularly in the Greek case, is inextricably linked to long-term strategic planning and management of the entire supply chain. Despite the notable progress in increasing our understanding of the wine sector supply chain [17,18] or the components of the wine tourism as a complex system [19,20], to the authors’ knowledge, no recent papers have attempted to provide a thorough analysis of the intricate nature of the wine supply chain. It is upon this last point that the current paper focuses on. Given the intricacy of the entities involved in the wine sector, the present study aims to offer a framework for mapping end-to-end the wine supply chain, illustrating all the processes and stakeholders involved.

## 2. Materials and Methods

### 2.1. Mapping Supply Chain Theoretical Foundations

Supply chains are, by default, complex systems involving several processes, decision points, stakeholders and interactions. A visual representation of all these elements, also known as a supply chain map [21], is essential for unveiling this complexity. One of the most acknowledged mapping approaches was on the value stream [22], yet new scientific developments and different research objectives over time resulted in further methods. For example, the car sector focused more on material and information flow mapping aiming at a leaner production [23]. Another need that supply chain mapping fulfils is the simplified representation of the entities involved and their dynamics [24] at multiple levels, usually available in every supply chain [25]. Therefore, mapping holds a vital role in both developing and employing a strategy but does not provide all the essential information to manage the supply chain [26,27].

However, in the literature, less attention has been given to investigating food supply chains employing such mapping approaches, despite the high level of complexity in this sector. A seminal paper on this field by Taylor [28], recommends a value chain analysis approach through different stages, towards the improvement of the supply chain procedures. Specifically, the stages are described as follows: (i) recognise the business potential of the analysis; (ii) realise supply chain structure and choose a target value stream; (iii) investigate the discrete facilities through the supply chain; (iv) mapping the present state of the entire value chain; (v) explore the problems and opportunities end-to-end in the supply chain; (vi) map the entire chain future state map; (vii) create an approachable organisational context [28]. A similar mapping method has been employed in many cases, for example for greenhouse gas emission mitigation strategies [29], to reduce food waste losses and waste in supply chains [30], to analyse the supply chain strategy and to propose a research framework [31].

### 2.2. Data and Research Design

The data used in this work to support the wine supply chain mapping, include personal communication with executives of the Greek Ministry of Rural Development and Food, secondary sectoral data [3,32] and five expert interviews with key sectoral stakeholders, all members of the recently established “Wine Tourism Committee”. The latter is a working group founded in 2015 under the National Inter-Professional Organization of Wine and Vine [5] and comprises winemakers and executives that represent all six Regional Winemakers’ Associations; the Greek Wine Federation [7]; and the Central Union of Vine and Wine Producing Cooperative Organizations of Greece [33]. Supplementally, five open-ended interviews with other actors of the wine supply chain were also conducted (viticulturists; wine writers; retailers; hoteliers and restaurateurs; wine tour operators). The informants were selected due to their extensive experience in the field (i.e., purposive sampling method). Overall, the sample is representative of the entire country’s wine sector, including wine tourism.

The methodological approach combines two different techniques, typical supply chain mapping employing value stream mapping [22]—value chain analysis [28] and case study approach [34,35]. The value chain includes the complete activities of all the companies involved, while the value stream refers only to the certain element that adds value to a specific product/service of the supply chain. Combining both provides clear advantages. Practically, we have used both secondary and primary research tools under a four-step abstraction process [31]. The first step provides an overall understanding of the processes and stakeholders involved and, also, defines the objective of the mapping supported by desk research (e.g., scientific publications and sectoral reports). Based on this understanding, the second step drafts the supply chain structure maps. The third step incorporates the individual entities and their interactions throughout the chain. It also validates, finetunes and eventually completes the theoretical mapping, according to the feedback from the open-ended interviews. Lastly, in step 4, there is a reporting of the maps and the overall insights.

## 3. Results and Discussion

Food supply chains involve a certain level of complexity [36] yet, the wine sector and the wine supply chains seem to be even more complicated [37,38]. One of the key reasons lies in the fact that the wine sector involves both tangible and intangible aspects which are incorporated in a typical product supply chain and a services supply chain [12]. The former deals with grape production (The classic varieties in Greece are Agiorgitiko, Xinomavro, Moschofilero and Assyrtiko. There are several emerging varieties as well, including Aidani, Athiri, Debina, Kidonitsa, Kotsifali, Liatiko, Limnio, Limniona, Malagousia, Mandilaria, Mavrodaphne, Mavrotragano, Monemvasia, Muscat of Alexandria, Robola, Roditis, Savatiano, Vidiano, Vilana, White Muscat [5]). White wines aredominant, while red wines represent only one third of the country’s total wine production. There no major differences with respect to the wine making process, the distribution and the retailing of bottled wine [5]. The latter deals with the broader concept of the experience related to wine, the dining sector, hospitality and wine tourism and all their relevant aspects [39].

Such a major differentiation dictates, as a first objective of mapping, the examination of the product supply chain, from grape to bottle and eventually to shelf. This mapping shall identify all the stages of the supply chain and their processes, achieving an initial simplification of the supply chain. Accordingly, the second mapping objective lies in the analysis of the wine dining and tourism supply chain, illustrating the system and network of all the stakeholders concerned along with their interdependencies [19]. Given the particularities of the services supply chains and the wide range of actors and businesses involved in the wine industry, the third mapping objective is to map the wine ecosystem from a holistic sustainability perspective [40].

### 3.1. From Grape to Shelf, Wine Supply Chain Mapping

**Figure 1:**
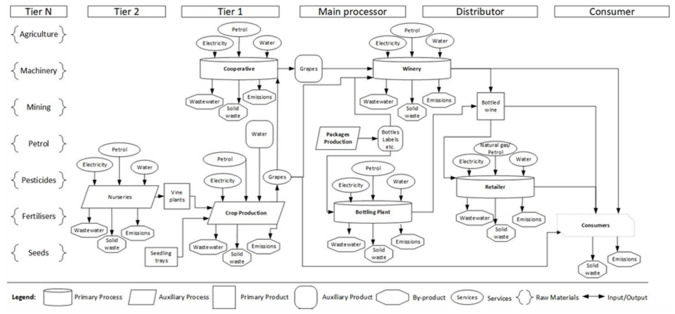
Wine supply chain, from grape to shelf.

The Wine Supply Chain—from grape to shelf—map (Figure 1) is a graphic illustration of the various processes, different types of products, raw materials and their respective input/output flows in every supply chain stage of the wine industry. The early production stages (Tier N) refer to the raw materials involved in both grape and wine production. Starting from the primary production, the analysis maps basic inputs from fertilisers and pesticides to equipment, petrol and all the essential resources for a proper production operation. The following stages (Tier 2 and Tier 1) depict the production phases, from nurseries developing the vineyard plans, crop production that provides the grapes and farm organisations managing the postharvest treatment. The next stages refer to the processing and distribution. The main activities here are winemaking and bottling, involving everything that transforms grapes into bottled wine. The distribution refers to the stage at which the final product is delivered to the consumer, i.e., the final stage of the supply chain. The map shows the processes, products and by-products (both primary and auxiliary), the services and their direction (input/output), which is consistent with similar mapping approaches [31].

A wine supply chain map under this angle contributes to a more effective analysis of the supply chain, for example concerning sustainability performance or resource efficiency assessment. The structure of the supply chain and the processes with the respective input/out could be used in a hotspot analysis towards the identification of resource efficiency issues [41,42]. From a sustainability perspective, the wine supply chain map (Figure 1) could serve as a fundamental stage in the overall understanding of the supply chain under investigation [43,44]. In fact, several mapping methodologies and sustainability assessment tools—e.g., SAFA (Sustainability Assessment of Food and Agriculture Systems) developed by FAO [45]—demand a similar approach and mapping as a prerequisite of their analysis.

### 3.2. Wine Value Chain Mapping and Wine Tourism as a System

**Figure 2:**
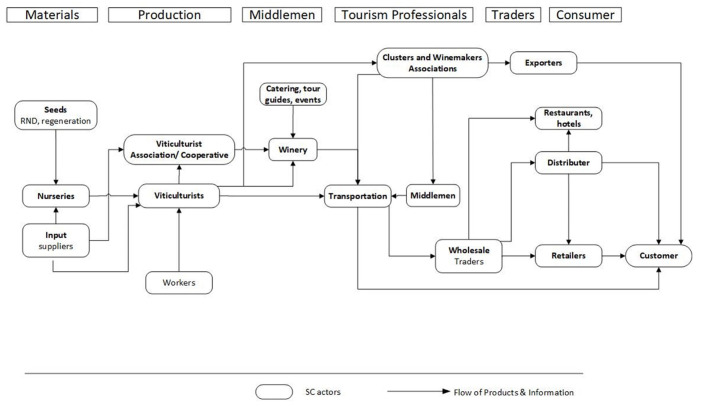
Wine value chain.

The wine value chain (Figure 2) illustrates all the stages and actions necessary to bring grapes from seed, through the entire supply chain (involving processing, bottling, storing, transportation and the input of various stakeholder services, i.e., winemakers, caterers and retailers) to the final consumers. The important part is introducing the wine experience and all the relevant stakeholders, including tourism professionals, accommodation providers and restaurateurs. Beginning with the materials stage, the key player here is the nursery, withmain inputs such as seeds and other supplies also included. The production follows, with the main actors being the viticulturists, labourers and cooperatives/associations, while the end product in this stage are the fresh grapes. The next stage involves middlemen, winemakers (including their clusters and associations), wineries and transportation actors. Beyond this stage, the product flows involve mainly wine (and a small percentage of table grapes). Most importantly, after the production of wine at this stage, there is an introduction of “wine experience” stakeholders and activities, such as caterers, tour guides, hoteliers and restaurateurs. The following stage involves key actors the traders, exporters, wholesalers, distributors and retailers—including outlets like dining providers and so on. The final stage concerns the consumer. Nevertheless, the goal of the mapping is to unwrap the complicated configuration of the supply chain via the demonstration of all its actors and stakeholders. A key element in this procedure is to reveal the way all these players are interrelated through the exchange of products and information. Correspondingly to value chain analysis [46,47], the current work entailed a certain value stream and, therefore, the flow of products and information suggests a key component of this map. The objective here is to take a snapshot of the supply chain towards a better understanding of its vital characteristics [28,31].

From a research design angle, the current mapping contributes to revealing significant bottlenecks of supply chain information flows, which result in major problems concerning supply chain performance. For example, a study in the Greek sustainable citrus sector suggested collaboration issues among key stakeholders [36] and in a similar way a study in the Dutch organic tomato supply chain unveiled the dominance of certain stakeholders over others [48]. The core of the mapping offered in this section is to develop a systemic map of the value chain through the methodical consideration of every element about the wine supply chain considering as well wine tourism as a system [39]. Consequently, the complicated nature of the products and information flows unveiled, leading to specific interventions that maximise the overall supply chain performance with clear benefits for all the stakeholders.

### 3.3. Wine Supply Chain and Stakeholders, a Holistic Mapping

**Figure 3:**
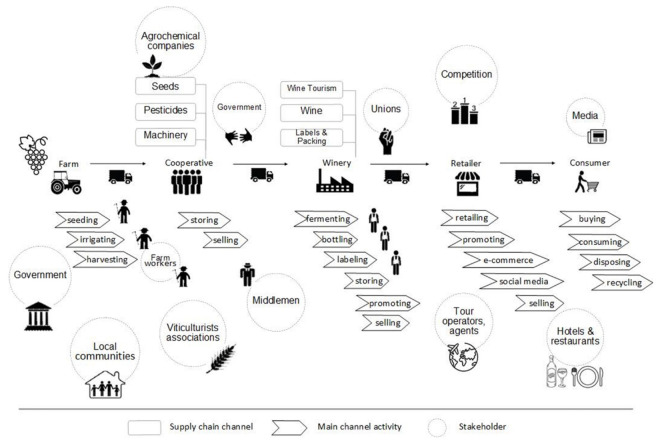
Wine Supply Chain and Stakeholders adopted from Busse, Schleper, Weilenmann, and Wagner [49]; Anastasiadis, Apostolidou and Michailidis [31].

The Wine Supply Chain and Stakeholders map (Figure 3) demonstrates all the processes and stakeholders implicated in the wine supply network, incorporating their interactions, critical points, the flow of products and information. In fact, it is an updated and more thorough value chain map (see previous section Figure 2) centred on the stakeholders. Figure 3 shows more clear associations of all stakeholders, for example, government, local communities, unions, tour operators, agents and hoteliers/restaurateurs. In particular, the first and second stages concern the production of fresh grapes and the basic winemaking process; therefore, the key players comprise viticulturists, associations, workers, local communities, government, agrochemical companies and middlemen. The key activities include seeding, irrigating, harvesting, storing and selling. Processing is the following stage, having as main actors the unions and main activities fermenting, bottling, labelling, storing, promoting and selling. This stage concludes with the production of wine and, therefore, the “wine experience”-related activities and stakeholders are also introduced. These are tour operators, agents, caterers, hoteliers, restaurateurs which are also involved in the following stage, i.e., the retailing. The key activities in both stages involvepromoting and selling. The final stage is—of course—the wine consumer. Here, the main actor is the media and the core activities include buying, consuming, disposing and recycling. The map depicted in Figure 3 illustrates the dynamics among the supply chain actors and the various processes throughout the supply chain. Consequently, it provides valuable input for further assessment towards improved supply chain performance.

Stakeholders hold a significant position in every supply chain and their involvement, specifically in the complicated food supply chain and their management, is essential. Previous studies suggest their pivotal role in sharing information towards food safety [50] and better performance of the food chain [36], in establishing new traceability systems and effective approaches such as consumer-centricity [51] and promoting sustainability and circular economy [52]. Supply chain re-configurations based on a better knowledge of the stakeholders’ role and interdependencies is the essence of every research framework. Figure 3 visualises all these vital elements concerning the Greek wine supply chain, providing thus a valuable tool for targeted research and effective interventions.

As already mentioned in previous studies [31] that have employed similar mapping techniques in different food chains, the combination of the three maps provides a very strong foundation in designing an effective research framework. Such a framework shall consist of all the necessary background information to set up the research objectives and to define the research directions. In the case of the Greek wine industry, using these maps future research could involve a sustainability assessment, using, for example, hot spot analysis [41]. A particular interest in this analysis could be resource efficiency and social sustainability issues [47], as illustrated in the proposed maps. Another recommendation could be a systematic analysis of the wine sector, given the dimension of the wine experience and all the respective stakeholders involved [53]. One of the particularities of the specific product is the involvement of the tourism stakeholders and actors around the consumption of wine including caterers, hoteliers, restaurateurs, wine festivals, shows and other gastronomic event providers, and everything related to the overall wine experience. This results in a complicated environment that includes both product and services supply chains. Therefore, a holistic approach is essential in understanding the nature of such complicated networks. Such a perspective provides tangible advantages compared to a myopic view focusing only on specific elements.

## 4. Conclusions

This study offers three maps covering the perquisites for the development of a powerful research framework in great depth. They reveal the bigger picture of the complex Greek wine supply chain and provide information concerning all the interdependencies among stakeholders. These insights consist of a fundamental first step towards drafting a powerful research framework. Beginning with the production from grape to shelf map (Figure 1), it depicts its process and gives further information about their input-output indicating crucial areas to be evaluated from a sustainability assessment point of view. Following, the wine value chain map (Figure 2) illustrates the complex flows of products and information indicating potential bottlenecks. The last map, wine supply chain and stakeholders (Figure 3), focuses on the actors and gives additional information about the entire wine industry. The combination of all maps provides an in-depth understanding of the Greek wine sector. The present work adds value by offering a more comprehensive understanding of the supply chain. The proposed approach has managerial and practical implications that extend beyond the building of an influential framework as outlined above. Any meaningful agri-food supply chain configuration should be made based on a similar mapping.

Moreover, given the multiple challenges related to the economic and the pandemic crisis, enhancing the resilience of the Greek wine industry remains a major issue, strongly connected with several environmental, social and political aspects of the sector [16]. Future research that could shed light in this respect requires extensive mapping of the stakeholders and processes involved, along with their interactions. The present study provides the first step towards this direction given that the significant role of stakeholders, as represented in the proposed maps, could offer considerable insights. The holistic approach that examines the Greek wine supply chain as a system is a significant insight and among the highest research priorities. Viewing all three maps under this angle reveals the vital role of the ‘wine experience’ for the entire system. Future research should focus on that direction, considering both tangible and intangible features of the wine supply chain.
